# Numerical simulation of a nonlinear hepatitis B virus mathematical model using the Dickson collocation technique

**DOI:** 10.1038/s41598-025-31826-0

**Published:** 2025-12-27

**Authors:** Atallah El-Shenawy, Mohamed El-Gamel, Mostafa Abouelsaid

**Affiliations:** 1 Department of Mathematics & Engineering Physics, Mansoura University, Mansoura, Egypt; 2 Engineering Mathematics, Physics, Science Department, Damietta University, Damietta, Egypt

**Keywords:** Hepatitis B virus, Dickson polynomial, Collocation, Mathematical biology, Nonlinear system, Newton algorithm, IVPs, Diseases, Mathematics and computing

## Abstract

This work introduces an innovative nonlinear model of hepatitis B virus (HBV) dynamics, emphasizing the utilization of the Dickson collocation method for numerical simulation. Our method, in contrast to conventional techniques, adeptly tackles the complex interactions between the virus and the host’s immune response using a system of ordinary differential equations (ODEs). We present a transformation of the ordinary differential equations into a nonlinear system of algebraic equations, facilitating the determination of unknown coefficients in a truncated Dickson polynomial series. The novel implementation of the Newton algorithm for addressing this nonlinear system improves computing efficiency and convergence rate. Our findings indicate that the Dickson collocation method provides highly precise approximations and surpasses traditional artificial neural network models regarding convergence rate and computational efficiency. This study highlights the Dickson collocation approach as an effective instrument for simulating intricate biological systems, offering valuable insights into HBV dynamics and laying the groundwork for future research.

## Introduction

In the 1960s, Dr. Baruch Blumberg discovered hepatitis B surface antigen, this opens the path to investigate its pathogenesis, prevention, and treatment. The Hepatitis B virus is a member of an immense family called Hepadnaviridae, a non-cytopathic DNA virus that produces an immune response affecting the liver with different presentation^1^. Africa and the western Pacific are known as the regions with high prevalence due to reduced birth vaccination. Instantly, $30\%$ of the population displays HBV evidence of either positive or previous infection which is why, HBV infection becomes a significant health concern^2^. Despite the extensive vaccination programs and effective medicine that targets suppressing the viral load^3^, chronic HBV infection remains a serious health problem affecting more than 290 million people worldwide, with an estimated global prevalence of about $4\%$, with different allocation and prevalences about the geographical area, the distribution of vaccination, and the risk factors^4^. It is associated with more than 800, 000 deaths per year, additionally, patients face a considerable risk of complications^5^. The World Health Organization (WHO) set a plan to eradicate viral hepatitis by 2030^6^. Regarding HBV structure it is classified as an enveloped virus with a partially double-stranded, circular DNA genome within the family of Hepadnaviridae and is characterized as a strictly species- and tissue-specific virus^7^.

In order to examine transmission patterns, assess intervention options, and forecast illness progression, academics and public health authorities now rely heavily on mathematical modeling to understand the dynamics of infectious diseases. The intricate relationships between susceptible, infected, and recovered persons must be adequately captured by nonlinear mathematical models due to the intricacy of HBV transmission. These models extensively analyze the factors impacting infection rates, recovery patterns, and the efficiency of vaccination and treatment options. A more realistic depiction of biological realities in disease transmission, such as saturation effects and host immunity consequences, is made possible by the inclusion of nonlinear components.

The nonlinear hepatitis B virus model is considered as follows:

Let $\phi (\zeta )$, $\epsilon$ and $\psi$ be the uninfected, infected and virus-free factors, then the nonlinear first order ordinary differential equations can be written as follows:

1 $$\begin{aligned} \phi '(\zeta )&=\theta _1 - \theta _2\phi (\zeta ) - \theta _3\psi (\zeta )\phi (\zeta ), \nonumber \\ \chi '(\zeta )&= \theta _3\psi (\zeta )\phi (\zeta ) - \theta _4\chi (\zeta ), \nonumber \\ \psi '(\zeta )&= \theta _5\chi (\zeta ) - \theta _6\psi (\zeta ), \end{aligned}$$

and their initial values at $\zeta = 0$ are given by

2 $$\begin{aligned} \phi (0)&= \varrho _1, \nonumber \\ \chi (0)&= \varrho _2, \nonumber \\ \psi (0)&= \varrho _3. \end{aligned}$$

The biological meaning of coefficients $\theta _1-\theta _6$ is summarized in Table 1^8^.

**Table 1:** The biological meaning of the coefficients in the nonlinear hepatitis B virus model 1 .

Coefficient	Biological meaning
$\theta _1$	Production rate according to innovative targeted cells.
$\theta _2$	Death of targeted cells.
$\theta _3$	Infection rate of innovative targeted cells.
$\theta _4$	Death rate of infected cells.
$\theta _5$	Rate of production of virions based on infected cells.
$\theta _6$	Clearance rate of virions free.

Differential equations not only describe important physical and engineering phenomena^9^ but also the epidemic behavior^10^. Computational techniques for numerically solving ordinary and partial differential equations (ODEs and PDEs) include several methods aimed at approximating solutions when analytical approaches are impractical^11–13^. These techniques encompass finite difference methods, which estimate derivatives by difference equations, and finite element approaches, which entail subdividing the domain into smaller, more manageable components (elements) and developing localized solutions. Alternative methodologies, like spectral methods, utilize orthogonal polynomials to provide elevated precision, especially for problems characterized by smooth solutions. Each method possesses distinct advantages and is chosen according to the particular attributes of the issue, such as boundary conditions, solution smoothness, and computational efficiency.

The novelty of this study can be summarized in the following contribution points:

- It is the first time to solve the HBV nonlinear model with spectral methods using Dickson polynomials as bases functions.
- Our methodology involves transforming the ordinary differential equations into a nonlinear system of algebraic equations. This transformation facilitates the identification of unknown coefficients within a truncated Dickson polynomial series, which is a novel approach in this context.
- The collocation–Newton framework enhances precision and reduces computational cost compared to existing approaches. These contributions introduce a powerful methodological tool for exploring biological complexity in infection modeling.
- Our findings demonstrate that the Dickson collocation method yields highly accurate approximations, outperforming conventional artificial neural network models in terms of convergence rate and computational efficiency.
- This study establishes the Dickson collocation method as a powerful tool for simulating complex biological systems, paving the way for future explorations into HBV dynamics and related fields.

Collocation techniques are a popular numerical technique for solving differential equations, wherein the solution is approximated by a linear combination of basis functions, and the differential equation is enforced at a designated collection of discrete locations, known as collocation points. This method has multiple advantages, including the utilization of basis functions, which may lead to improved accuracy, particularly for problems with complex geometries or boundary conditions. Moreover, collocation approaches may exhibit greater computing efficiency compared to traditional methods, as they often necessitate fewer degrees of freedom to attain the desired precision^14,15^. Their capacity to manage diverse boundary conditions and their relevance to both linear and nonlinear problems further augment their usefulness in mathematical modeling and simulation. Various bases functions have been merged with the collocation method to solve all types of differential and integral equations, for example B-spline functions^11,16,17^, Sinc^18,19^, Chebyshev^10^, Jacobi^20^ and Bessel polynomials^21^. And recently many authors applied Dickson polynomials due to their efficiency and high accuracy in solving these types of problems^22,23^. The Dickson collocation technique also has been implemented to solve and simulate important physical and biological modes, for examples the nonlinear system regarding pollution of a system of lakes as in^24^, SIR fractional model of the distribution of computer viruses in^25^. On the other hand, the method is developed to solve not only models related to differential equations with integer order derivatives but also the delay integro-differential equations^26^ and fractional functional differential equations in^27^. The mentioned examples emphasize the efficiency and the variety of the Dickson collocation technique for solving many applied and engineering problems.

This document is structured into six sections to facilitate a comprehensive study of Dickson polynomials and their applications. Section Introduction provides the introduction, delineating the rationale and aims of the investigation. Section Basic definitions of Dickson polynomials delineates the fundamental definitions of Dickson polynomials, establishing a foundation for succeeding discussions. Section Convergence analysis truncated Dickson series examines the convergence analysis of truncated Dickson series, offering insights into their behavior and efficacy. Section Dickson operational matrices collocation method (DOMCM) presents the Dickson operational matrices collocation method (DOMCM), elucidating its derivation and benefits when dealing with the HBV nonlinear mathematical model. Section Results and simulations delineates the results and simulations, illustrating the efficacy of the offered methodologies via diverse numerical examples. Section Conclusions finally terminates the work by summarizing the principal findings and proposing avenues for future investigation.

## Basic definitions of Dickson polynomials

### Definition 1

The 2^nd^ order Dickson ordinary differential equation is defined by^26^

$$\begin{aligned} (\varsigma ^2-4\nu )\vartheta ^{\prime \prime } + \varsigma \vartheta ^{\prime }-n\vartheta = 0,\qquad n = 0,1,\cdots \end{aligned}$$

where $\nu \in \mathbb {R}$ is the Dickson parameter.

### Definition 2

The first kind Dickson polynomial $\mathfrak {D}_{n}(\zeta ,\nu )$ is the solution of the Dickson ordinary differential equation and can be expressed

3 $$\begin{aligned} \mathfrak {D}_{n}(\zeta ,\nu ) = \sum _{i=0}^{\lfloor \frac{n}{2}\rfloor }{\frac{n}{n-i}\left( {\begin{array}{c}n-i\\ i\end{array}}\right) (-\nu ^i)\zeta ^{n-2i}}, \qquad \zeta \in (-\infty ,\infty ). \end{aligned}$$

### Lemma 1

*Dickson polynomials have the following properties* ^26,28^ *:*

$$\begin{aligned} \mathfrak {D}_{n}(\zeta ,\nu ) = \zeta \mathfrak {D}_{n-1}(\zeta ,\nu ) - \nu \mathfrak {D}_{n-2}(\zeta ,\nu ). \end{aligned}$$

with $\mathfrak {D}_{0}(\zeta ,\nu ) = 2,$ $\mathfrak {D}_{1}(\zeta ,\nu ) = \zeta .$ Table 2 tabulated the first terms of Dickson polynomials up to $n=5$:

**Table 2:** The first terms of Dickson polynomials.

*n*	$\mathfrak {D}_{n}(\zeta ,\nu )$
0	$\mathfrak {D}_{0}(\zeta ,\nu ) = 2$
1	$\mathfrak {D}_{1}(\zeta ,\nu ) = \zeta$
2	$\mathfrak {D}_{2}(\zeta ,\nu ) = \zeta ^2 - 2\nu$
3	$\mathfrak {D}_{3}(\zeta ,\nu ) = \zeta ^3 - 3\nu \zeta$
4	$\mathfrak {D}_{4}(\zeta ,\nu ) = \zeta ^4 - 4\nu \zeta ^2 + 2\nu ^2$
5	$\mathfrak {D}_{5}(\zeta ,\nu ) = \zeta ^5 - 5\nu \zeta ^3 + 5\nu ^2\zeta$

The value of $\nu$ reduces the Dickson polynomial to one of four different types of well-known polynomials. Table 3 states the different types with their corresponding values of $\nu$.

**Table 3:** Different polynomials derived from Dickson polynomial.

No	$\nu$	Polynomial
1	$-1$	Pell-Lucas polynomials
2	0	Power polynomials
3	1	First kind Chebyshev polynomials
4	2	Ferma-Lucas polynomials

## Convergence analysis truncated Dickson series

Truncated Dickson series gives a good approximation of the continuous functions.

### Definition 3

$$\varepsilon _s = {\left\{ \begin{array}{ll} 0.5, \qquad & j=0,\\ 1, \qquad & s\ge 1. \end{array}\right. }$$

The following lemmas are essential in proving the error bound of the truncated Dickson series approximation. All proofs can be found in detail in^22^.

### Lemma 2

*If* $\varpi \in [0,1],$ *then* $\forall i \in \textbf{N}$ *the Dickson polynomial is bounded above by:*

$$\begin{aligned} \big | \mathfrak {D}_{\zeta ,\nu }(\varsigma )\big |\le 2\big (1+\sqrt{1+|\nu |}\big )^i. \end{aligned}$$

### Lemma 3

*Let the function* $\varpi (\zeta )$ *has bounded derivatives at* $=0$*,* $\big |\varpi ^{(k)}(0)\big |\le L^k$*, and* $\varpi (\zeta ) = \sum _{i=0}^{\infty }{\alpha _i\mathfrak {D}_{i,\nu }(\varsigma )},$ *then*

$$\begin{aligned} |\alpha _i|\le \frac{C^i\cosh {\big (2L\sqrt{|\nu |}\big )}}{\Gamma {(i+1)}}. \end{aligned}$$

### Theorem 1

Assume that the function $\varpi (\zeta )$ *has bounded derivatives at* $=0$*,* $\big |\varpi ^{(k)}(0)\big |\le L^k$*, and* $\varpi (\zeta ) = \sum _{i=0}^{\infty }{\alpha _i\mathfrak {D}_{i,\nu }(\zeta )}.$ *Let* $\varpi _N(\zeta )=\sum _{i=0}^{N}{\alpha _i\mathfrak {D}_{i,\nu }(\zeta )}$ *be the best approximation of* $\varpi (\zeta )$ *in the space* $\mathbb {S} = \text {Span}\{\mathfrak {D}_{0,\nu }(\zeta ),\mathfrak {D}_{1,\nu }(\zeta ),\cdots ,\mathfrak {D}_{N,\nu }(\zeta )\},$ *then the truncation error bound is*

$$\begin{aligned} \big |\varpi (\zeta )-\varpi _N(\zeta ) \big |\le \frac{2e^{CH}\left( CH\right) ^{N+1}\cosh {\left( 2LH\right) }}{\Gamma {(N+2)}}. \end{aligned}$$

### Proof

$$\begin{aligned} \big |\varpi (\zeta )-\varpi _N(\zeta ) \big |= & \big | \sum _{i=N+1}^{\infty }{\alpha _i\mathfrak {D}_{i,\nu }(\varsigma )}\big |,\\\le & \sum _{i=N+1}^{\infty }{\big |\alpha _i\big |\big |\mathfrak {D}_{i,\nu }(\zeta )\big |},\\\le & \sum _{i=N+1}^{\infty } {\frac{C^i \cosh {(2L\sqrt{|\nu |})}}{\Gamma {(i+1)}}\times 2(1+\sqrt{1+|\nu |})},\\\le & 2\cosh {(2L\sqrt{|\nu |})}\sum _{i=N+1}^{\infty }{\frac{\left[ C(1+\sqrt{1+|\nu |})\right] ^i}{\Gamma {(i+1)}}}. \end{aligned}$$

Let $$H = 1+\sqrt{1+|\nu |}$$, then,

$$\begin{aligned} \big |\varpi (\zeta )-\varpi _N(\zeta ) \big |\le & 2\cosh {(2L\sqrt{|\nu |})} \sum _{i=N+1}^{\infty }{\frac{\left[ CH\right] ^i}{{i!}}},\\\le & 2e^{CH}\cosh {(2L\sqrt{|\nu |})}\left[ 1-\frac{\Gamma {(N+1,CH)}}{\Gamma {(N+1)}} \right] ,\\\le & 2e^{CH}\cosh {(2L\sqrt{|\nu |})}\int _{0}^{CH}{\eta ^Ne^{-\eta }d\eta },\\\le & 2e^{CH}\cosh {(2L\sqrt{|\nu |})}\frac{\left[ CH\right] ^{N+1}}{{(N+1)!}},\\\le & \frac{2e^{CH}\cosh {(2L\sqrt{|\nu |})}\left[ CH\right] ^{N+1}}{(N+1)!}. \end{aligned}$$

## Dickson operational matrices collocation method (DOMCM)

### Construction of the discrete system

Assume that the knot points $\zeta _k, k=0,1,\cdots , N$ be $N+1$ points in the solution domain [0, 1]. Let the solution of the hepatitis B virus model (1); the three unknown functions $\phi (\zeta ), \chi (\zeta )$ and $\psi (\zeta )$ can be expressed by means of truncated Dickson polynomials in the form:

4 $$\begin{aligned} \phi (\zeta _k)&= \sum _{i=0}^M {v_i\mathfrak {D}_{i}(\zeta _k,\nu )} \nonumber \\ \chi (\zeta _k)&= \sum _{i=0}^M {h_i\mathfrak {D}_{i}(\zeta _k,\nu )} \end{aligned}$$

5 $$\begin{aligned} \psi (\zeta _k)&= \sum _{i=0}^M {s_i\mathfrak {D}_{i}(\zeta _k,\nu )} \end{aligned}$$

The approximate solutions will be determined by solving a nonlinear system of equations of the unknown coefficients $\{v_i\}_{i=0}^{M}, \{h_i\}_{i=0}^{M}\}$ and $\{s_i\}_{i=0}^{M}$. The following theorem helps in constructing the discrete system for the nonlinear hepatitis B virus model (1).

#### Theorem 2

*Assume that the truncated series (*4*) are the solutions of nonlinear hepatitis B virus model* *(*1*-*2*), then the unknown coefficients* $\{v_i\}_{i=0}^{M}, \{h_i\}_{i=0}^{M}\}$ *and* $\{s_i\}_{i=0}^{M}$ *are determined by solving the following nonlinear system of algebraic equations in augmented matrix form*

6 $$\begin{aligned} [\boldsymbol{\Delta (\Gamma )}] = 0. \end{aligned}$$

#### Proof

If the approximate solution of (1-2) are the truncated series in (4), then the first derivatives can be calculated directly as follows:

7 $$\begin{aligned} \phi ^\prime (\zeta )&= \sum _{i=0}^M {v_i\mathfrak {D}^\prime _{i}(\zeta ,\nu )}, \nonumber \\ \chi ^\prime (\zeta )&= \sum _{i=0}^M {h_i\mathfrak {D}^\prime _{i}(\zeta ,\nu )}, \end{aligned}$$

8 $$\begin{aligned} \psi ^\prime (\zeta )&= \sum _{i=0}^M {s_i\mathfrak {D}^\prime _{i}(\zeta ,\nu )}. \end{aligned}$$

By substituting the approximate solutions and their first derivatives in the nonlinear system of equations we obtain:

9 $$\begin{aligned} \sum _{i=0}^M {v_i\mathfrak {D}^\prime _{i}(\zeta ,\nu )}&= \theta _1 - \theta _2 \left( \sum _{i=0}^M {v_i\mathfrak {D}_{i}(\zeta ,\nu )} \right) - \theta _3\left( \sum _{i=0}^M {s_i\mathfrak {D}_{i}(\zeta ,\nu )} \right) \cdot \left( \sum _{i=0}^M {v_i\mathfrak {D}^\prime _{i}(\zeta ,\nu )} \right) , \nonumber \\ \sum _{i=0}^M {h_i\mathfrak {D}^\prime _{i}(\zeta ,\nu )}&= \theta _3\left( \sum _{i=0}^M {s_i\mathfrak {D}_{i}(\zeta ,\nu )} \right) \left( \sum _{i=0}^M {v_i\mathfrak {D}_{i}(\zeta ,\nu )} \right) - \theta _4 \left( \sum _{i=0}^M {h_i\mathfrak {D}_{i}(\zeta ,\nu )} \right) , \nonumber \\ \sum _{i=0}^M {s_i\mathfrak {D}^\prime _{i}(\zeta ,\nu )}&= \theta _5 \left( \sum _{i=0}^M {h_i\mathfrak {D}_{i}(\zeta ,\nu )} \right) - \theta _6 \left( \sum _{i=0}^M {s_i\mathfrak {D}_{i}(\zeta ,\nu )} \right) . \end{aligned}$$

After applying the collocation points, we easily obtain the discrete system of our problem.

### Reduction to matrix form

To simplify the computational process, the discrete system will be written in matrix form with the help of Dickson operational matrices as follows:

#### Lemma 4

*The truncated series expansions in Eq. (*4*) can be expressed in the following matrix form:*

10 $$\begin{aligned} \phi (\zeta )&\approx \mathbf {R(\zeta )J(\nu ) V}, \nonumber \\ \chi (\zeta )&\approx \mathbf {R(\zeta ) J(\nu ) H}, \nonumber \\ \psi (\zeta )&\approx \mathbf {R(\zeta ) J(\nu ) S}, \end{aligned}$$

*where*

$$\textbf{V} = [v_0, v_1, \dots , v_M]^T, \quad \textbf{H} = [h_0, h_1, \dots , h_M]^T, \quad \textbf{S} = [s_0, s_1, \dots , s_M]^T,$$

*are the unknown coefficients vectors of the three functions* $\phi (\zeta ), \chi (\zeta )$ *and* $\psi (\zeta )$*, respectively. And*

$$\begin{aligned} \textbf{R}(\zeta ) = [1, \zeta , \zeta ^2, \dots , \zeta ^M]^T, \end{aligned}$$

*is collocation points matrix,* $\mathbf {J(\nu )}$ *has two cases:*

If M is even

$$\small \mathbf {J^T(\nu )} = \begin{pmatrix} 2 & 0 & 0 & 0 & \dots & 0 \\ 0 & \frac{1}{1}\left( {\begin{array}{c}1\\ 0\end{array}}\right) (-\nu )^0 & 0 & 0 & \dots & 0 \\ \frac{2}{1}\left( {\begin{array}{c}1\\ 1\end{array}}\right) (-\nu )^1 & 0 & \frac{2}{2}\left( {\begin{array}{c}2\\ 0\end{array}}\right) (-\nu )^0 & 0 & \dots & 0 \\ 0 & \frac{3}{2}\left( {\begin{array}{c}2\\ 1\end{array}}\right) (-\nu )^1 & 0 & \frac{3}{3}\left( {\begin{array}{c}3\\ 0\end{array}}\right) (-\nu )^0& \dots & 0 \\ \vdots & \vdots & \vdots & \vdots & \ddots & \vdots \\ \frac{M}{M/2)}\left( {\begin{array}{c}M/2\\ M/2\end{array}}\right) (-\nu )^{M/2} & 0 & \frac{M}{(M/2)+1}\left( {\begin{array}{c}(M/2)+1\\ (M/2)-1\end{array}}\right) (-\nu )^{(M/2)-1} & 0 & \dots & \frac{M}{M}\left( {\begin{array}{c}M\\ 0\end{array}}\right) (-\nu )^0 \\ \end{pmatrix},$$

if M is odd

$$\small \mathbf {J^T(\nu )} = \begin{pmatrix} 2 & 0 & 0 & 0 & \dots & 0 \\ 0 & \frac{1}{1}\left( {\begin{array}{c}1\\ 0\end{array}}\right) (-\nu )^0 & 0 & 0 & \dots & 0 \\ \frac{2}{1}\left( {\begin{array}{c}1\\ 1\end{array}}\right) (-\nu )^1 & 0 & \frac{2}{2}\left( {\begin{array}{c}2\\ 0\end{array}}\right) (-\nu )^0 & 0 & \dots & 0 \\ 0 & \frac{3}{2}\left( {\begin{array}{c}2\\ 1\end{array}}\right) (-\nu )^1 & 0 & \frac{3}{3}\left( {\begin{array}{c}3\\ 0\end{array}}\right) (-\nu )^0& \dots & 0 \\ \vdots & \vdots & \vdots & \vdots & \ddots & \vdots \\ 0& \frac{M}{\big \lceil \frac{M}{2} \big \rceil } \left( {\begin{array}{c}\big \lceil \frac{M}{2} \big \rceil \\ \left\lfloor \frac{M}{2} \right\rfloor \end{array}}\right) (-\nu )^{\left\lfloor \frac{M}{2} \right\rfloor } & 0& \frac{M}{\big \lceil \frac{M}{2} \big \rceil +1} \left( {\begin{array}{c}\big \lceil \frac{M}{2} \big \rceil +1\\ \left\lfloor \frac{M}{2} \right\rfloor -1\end{array}}\right) (-\nu )^{\left\lfloor \frac{M}{2} \right\rfloor - 1} & \dots & \frac{M}{M} \left( {\begin{array}{c}M\\ 0\end{array}}\right) (-\nu )^0 \\ \end{pmatrix}.$$

#### Definition 4

The differentiation matrix $\textbf{C}$ is defined as follows:

$$\textbf{C} = \begin{pmatrix} 0 & 1 & 0 & 0 & \cdots & 0 \\ 0 & 0 & 2 & 0 & \cdots & 0 \\ 0 & 0 & 0 & 3 & \cdots & 0 \\ \vdots & \vdots & \vdots & \vdots & \ddots & \vdots \\ 0 & 0 & 0 & 0 & 0 & M \\ 0 & 0 & 0 & 0 & \cdots & 0 \\ \end{pmatrix}.$$

The next lemma is used to write the derivatives of the approximate solutions in terms of Dickson operational matrices:

#### Lemma 5

*Let* *K* *is an integer, then the K*_*th*_ *derivative of the the three functions* $\phi (\zeta ), \chi (\zeta )$ *and* $\psi (\zeta )$*, respectively are:*

11 $$\begin{aligned} \phi ^{(K)}(\zeta )&\approx \mathbf {R(\zeta )(C)^KJ(\nu )V}, \nonumber \\ \chi ^{(K)}(\zeta )&\approx \mathbf {R(\zeta )(C)^KJ(\nu )H}, \nonumber \\ \psi ^{(K)}(\zeta )&\approx \mathbf {R(\zeta )(C)^KJ(\nu )S}. \end{aligned}$$

The matrix form of the discrete system in Eq. (9) is obtained easily by replacing the unknown functions and their first derivatives by their corresponding matrix forms (10) and (11) with $K =1$, respectively.

12 $$\begin{aligned} \mathbf {R(\zeta )CJ(\nu )V}&= \theta _1 - \theta _2 \mathbf {R(\zeta )J(\nu ) V} - \theta _3\left[ \mathbf {R(\zeta ) J(\nu ) S}\right] \left[ \mathbf {R(\zeta )J(\nu ) V}\right] , \nonumber \\ \mathbf {R(\zeta )CJ(\nu )H}&= \theta _3\left( \mathbf {R(\zeta ) J(\nu ) S} \right) \left[ \mathbf {R(\zeta )J(\nu ) V} \right] - \theta _4 \left[ \mathbf {R(\zeta ) J(\nu ) H} \right] , \nonumber \\ \mathbf {R(\zeta )CJ(\nu )S}&= \theta _5 \left[ \mathbf {R(\zeta ) J(\nu ) H} \right] - \theta _6 \left[ \mathbf {R(\zeta ) J(\nu ) S} \right] . \end{aligned}$$

To enforce the approximate solutions to coincides with the true values at the initial points, the initial conditions (2) will be converted to matrix forms and inserted in the above system by replacing the first element in each vector of (12) as follows:

$$\begin{aligned} \phi (0)&= \sum _{i=0}^M {v_i\mathfrak {D}_{i}(0,\nu )}, \\ \chi (0)&= \sum _{i=0}^M {h_i\mathfrak {D}_{i}(0,\nu )}, \\ \psi (0)&= \sum _{i=0}^M {s_i\mathfrak {D}_{i}(0,\nu )}, \\ \end{aligned}$$

which easily written in matrix form

$$\begin{aligned} \mathbf {R(0) J(\nu ) V} -\varrho _1= & 0, \\ \mathbf {R(0) J(\nu ) H} - \varrho _2= & 0, \\ \mathbf {R(0) J(\nu ) S} - \varrho _3= & 0. \end{aligned}$$

The final matrix form of the $3M+3\times 3M+3$ nonlinear system of equation will be reduced as in Eq. (6), where

$$\boldsymbol{\Delta (\Gamma )} = \begin{pmatrix} \mathbf {R(0) J(\nu ) V} -\varrho _1\\ \mathbf {R(\zeta _k)CJ(\nu )V} - \theta _1 + \theta _2 \mathbf {R(\zeta _k)J(\nu ) V} + \theta _3\left[ \mathbf {R(\zeta _k) J(\alpha ) S}\right] \left[ \mathbf {R(\zeta _k)J(\nu ) V}\right] \\ \mathbf {R(0) J(\nu ) H} - \varrho _2 \\ \mathbf {R(\zeta _k)CJ(\nu )H} - \theta _3\left( \mathbf {R(\zeta _k) J(\nu ) S} \right) \left[ \mathbf {R(\zeta _k)J(\nu ) V} \right] + \theta _4 \left[ \mathbf {R(\zeta _k) J(\nu ) H} \right] \\ \mathbf {R(0) J(\nu ) S} - \varrho _3 \\ \mathbf {R(\zeta _k)CJ(\nu )S} - \theta _5 \left[ \mathbf {R(\zeta _k) J(\nu ) H} \right] + \theta _6 \left[ \mathbf {R(\zeta _k) J(\nu ) S} \right] \\ \end{pmatrix},$$

$$\zeta _k \in \big (0,1\big ]$$ and

$$\begin{aligned} \mathbf {\Gamma } = \big [\textbf{V},\textbf{H},\textbf{S}\big ]^T = \big [v_0, v_1, \dots , v_M,h_0, h_1, \dots , h_M,s_0, s_1, \dots , s_M\big ]^T. \end{aligned}$$

DOMCM scheme of solution with detailed steps are summarized in Algorithm 1.

**Algorithm 1:**
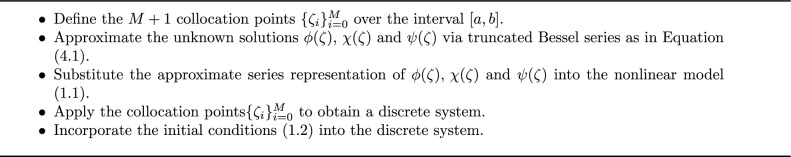
DOMCM scheme of solution.

### Solution of nonlinear system of equations

The obtained nonlinear solutions will be solved using the Newton iterative algorithm, the main steps are summarized in the Algorithm 2.

**Algorithm 2:**
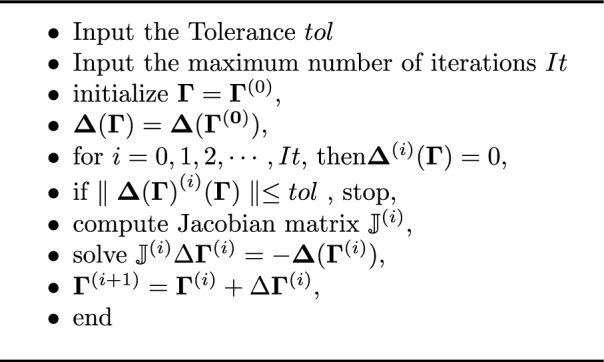
Newton iteration algorithm.

By computing the unknown coefficients $\mathbf {\Gamma }$, the approximate solutions can be found by substituting in Eq. (4).

## Results and simulations

This section presents the numerical findings derived from the application of the Dickson collocation technique to the nonlinear hepatitis B viral model. The goal of our research is to clarify the virus’s dynamic behavior under different parameter configurations and beginning conditions. We commence with an analysis of the numerical method’s convergence and computing efficiency, subsequently doing a thorough evaluation of the simulation results. The results emphasize significant trends in viral load, immune response, and the ramifications of treatment approaches. These studies aim to elucidate the intricate connections within the hepatitis B viral system, enhancing comprehension of its dynamics and guiding future research and therapy strategies. Assume that the parameters of the model (1-2) have the values tabulated in Table 4 as in^8^.

**Table 4:** Numerical values of the parameters in model (1-2).

Parameter	value
$\theta _1$	0.67
$\theta _2$	3.78
$\theta _3$	1
$\theta _4$	3.259
$\theta _5$	2.1
$\theta _6$	0.67
$\varrho _1$	5.5555
$\varrho _2$	1.1111
$\varrho _3$	6.3096

For the sake of comparison, the numerical solutions of the hepatitis B virus model by applying the different cases of Dickson collocation techniques are calculated.

### Residual error

#### Theorem 3

*Assume that* $\mathcal {R}_{1,N}(\zeta )$*,* $\mathcal {R}_{2,N}(\zeta )$*,* $\mathcal {R}_{3,N}(\zeta )$ *are the residual functions for the approximate solutions. Then, the following system related to the error problem is:*

13 $$\begin{aligned} \begin{aligned} \frac{d e_\phi }{d\zeta } + 3.78 e_\phi + e_\psi e_\phi + e_\psi \zeta _N + e_\phi \Psi _N&= -\mathcal {R}_{1M}, \\ \frac{d e_\chi }{d\zeta } - e_\psi e_\phi - e_\psi \zeta _N - e_\phi \Psi _N + 3.259 e_\chi&= -\mathcal {R}_{2M}, \\ \frac{d e_\psi }{d\zeta } - 2.1 e_\chi + 0.67 e_\psi&= -\mathcal {R}_{3M}. \end{aligned} \end{aligned}$$

*Where the error terms are given by:*

14 $$\begin{aligned} \begin{aligned} e_\phi (\zeta )&= \Phi (\zeta ) - \Phi _M(\zeta ), \\ e_\chi (\zeta )&= \chi (\zeta ) - \chi _M(\zeta ), \\ e_\psi (\zeta )&= \Psi (\zeta ) - \Psi _M(\zeta ). \end{aligned} \end{aligned}$$

#### Proof

^29^ Since the approximate solutions $\Phi _N(\zeta )$, $\chi _N(\zeta )$, and $\Psi _N(\zeta )$ satisfy the numerical model, we write the residual equations as:

15 $$\begin{aligned} \begin{aligned} \mathcal {R}_{1,M}(\phi )&= \frac{d \Phi (\zeta )}{d\zeta } + 3.78 \Phi (\zeta ) + \Psi (\zeta ) \Phi (\zeta ) - 0.67, \\ \mathcal {R}_{2,M}(\chi )&= \frac{d \chi (\zeta )}{d\zeta } - \Psi (\zeta ) \Phi (\zeta ) + 3.259 \chi (\zeta ), \\ \mathcal {R}_{3,M}(\psi )&= \frac{d \Psi (\zeta )}{d\zeta } - 2.1 \chi (\zeta ) + 0.67 \Psi (\zeta ). \end{aligned} \end{aligned}$$

Subtracting this system from the original system, employing the relations in (14), we obtain the error system (13).

### Stability and sensitive analysis

In our case we can perform the sensitivity analysis and the influence of the coefficients $\{\theta _1, \theta _2, \theta _3, \theta _4, \theta _5\}$ on the model by means of equilibrium points and linear stability as follows:

1. Firstly, we calculate the equilibrium points $(\phi , \chi , \psi )$ of the model by solving $\phi ^{\prime }(\zeta ) = 0,\, \chi ^{\prime }(\zeta ) = 0, \,\psi ^{\prime }(\zeta ) = 0$ The point is
  $$(\phi ,\chi ,\psi ) = \left( \frac{\theta _1}{\theta _2}, 0, 0 \right) .$$
2. Secondly, the Jacobian matrix evaluated at this equilibrium point is given by
  $$J = \begin{bmatrix} -\theta _2 & 0 & -\dfrac{\theta _1 \theta _3 }{\theta _2} \\ 0 & -\theta _4 & \dfrac{\theta _1 \theta _3 }{\theta _2} \\ 0 & \theta _5 & -\theta _6 \end{bmatrix}.$$
3. Thirdly, the characteristic equation corresponding to matrix *J* is
  $$-\theta _1 \theta _3 \theta _5 +\theta _2 \theta _4 \theta _6 + \left( {\theta _2 \theta _4} -\dfrac{\theta _1 \theta _3 \theta _5 }{\theta _2} +\theta _2 \theta _6 + \theta _4 \theta _6\right) \lambda + (\theta _2 + \theta _4 + \theta _6)\lambda ^2 + \lambda ^3 = 0.$$
  Recalling that the Routh–Hurwitz stability conditions for a cubic equation in the form
  $$\lambda ^3 + A_1 \lambda ^2 + A_2 \lambda + A_3 = 0$$
  are given by
  $$A_1> 0, \quad A_2> 0, \quad A_3> 0, \quad A_1 A_2 - A_3 > 0,$$
  where the coefficients of the characteristic equation corresponding to matrix *J* are
  $$A_1 = (\theta _2 + \theta _4 + \theta _6),$$
  $$A_2 = (\theta _2 \theta _4 -\dfrac{\theta _1 \theta _3 \theta _5 }{\theta _2}+ \theta _2 \theta _6 + \theta _4 \theta _6),$$
  $$A_3 =( -\theta _1 \theta _3 \theta _5 + \theta _2 \theta _4 \theta _6).$$
4. Finally, the stability condition can be deduced to be
  $$\theta _1> 0, \quad \theta _2> 0, \quad \theta _3 \ge 0, \quad \theta _4> 0, \quad \theta _5> 0, \quad \theta _6 > \frac{\theta _1 \theta _3 \theta _5}{\theta _2 \theta _4}.$$
  Where the $\theta$ values in Table 3
  $$\theta _1 = 0.67, \quad \theta _2 = 3.78, \quad \theta _3 = 1, \quad \theta _4 = 3.259, \quad \theta _5 = 2.1, \quad \theta _6 = 0.67$$
  achieve the above conditions.

By substituting the given values to calculate the eigenvalues, we obtain

$$\lambda = \{-11.1310,\;-3.4605,\;0.5729\}.$$

To estimate the sensitivity of eigenvalues with respect to the system parameters, we compute the derivatives

$$\frac{d\lambda }{d\theta _i}, \quad i = 1,2,\dots ,6.$$

In conclusion, the system eigenvalues show that the most sensitive parameter is $\theta _3$, as it significantly affects the eigenvalues. Parameters $\theta _2, \theta _4, \theta _5, \theta _6$ have moderate effects on the system stability.

### Numerical results

**Fig. 1:**
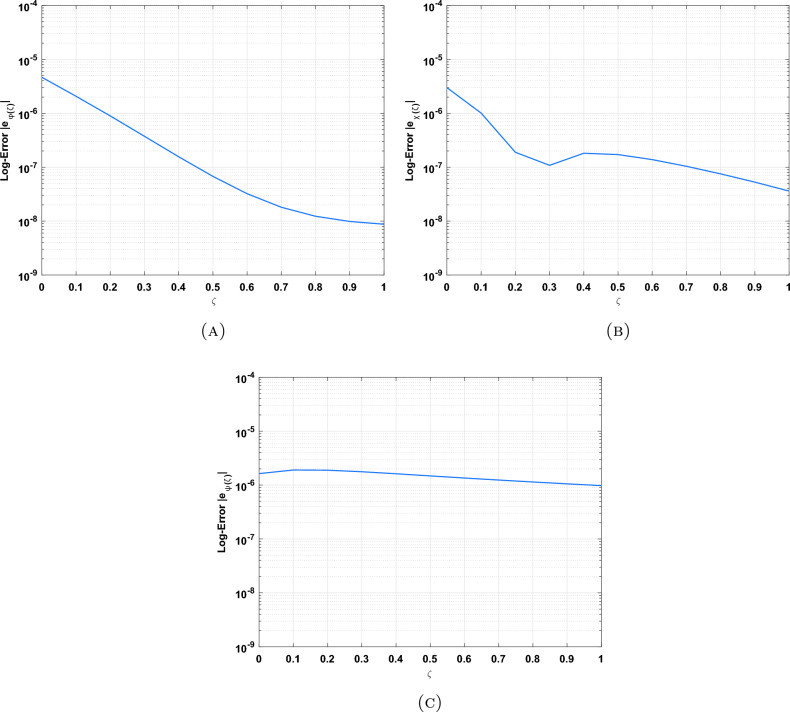
The log scale graph of the error functions values in Table 5.

To verify the theoretical results, we performed numerical simulations using MATLAB. The numerical solutions $\Phi _M(\zeta )$, $\chi _M(\zeta )$, and $\Psi _M(\zeta )$ were obtained using the Dickson polynomial method. The absolute error functions are calculated by solving the error system (13) and are tabulated in Table 5. Figure 1 shows the three error functions plotted on log-scale and the curves emphasize the accuracy of the proposed method.

**Table 5:** Error functions calculated at selected points$\zeta \in [0,1]$ in (13).

$\zeta$	$\Vert e_\phi (\zeta )\Vert$	$\Vert e_\chi (\zeta )\Vert$	$\Vert e_\psi (\zeta )\Vert$
0.0	4.657e-06	2.995e-06	1.632e-06
0.1	2.070e-06	1.009e-06	1.902e-06
0.2	8.941e-07	1.893e-07	1.887e-06
0.3	3.744e-07	1.084e-07	1.767e-06
0.4	1.561e-07	1.816e-07	1.620e-06
0.5	6.757e-08	1.716e-07	1.479e-06
0.6	3.223e-08	1.384e-07	1.351e-06
0.7	1.807e-08	1.040e-07	1.239e-06
0.8	1.230e-08	7.521e-08	1.141e-06
0.9	9.853e-09	5.283e-08	1.054e-06
1.0	8.763e-09	3.608e-08	9.768e-07

The absolute error between Runge–Kutta 4 and Dickson collocation method at $v=-1$ is displayed in Table 6.

**Table 6:** The absolute error between Runge–Kutta 4 and Dickson collocation method at $v=-1$.

$\zeta$	$|\textrm{error}\,\phi (\zeta )|$	$|\textrm{error}\,\chi (\zeta )|$	$|\textrm{error}\,\psi (\zeta )|$
0.0	0	0	0
0.1	6.572e-4	5.934e-4	1.192e-4
0.2	7.910e-5	6.858e-5	1.258e-5
0.3	2.247e-4	1.872e-4	3.039e-5
0.4	3.779e-6	2.990e-6	2.362e-8
0.5	1.882e-5	1.033e-5	2.231e-6
0.6	1.114e-5	2.834e-6	3.959e-6
0.7	6.909e-7	8.548e-8	6.016e-7
0.8	3.544e-8	6.323e-7	9.357e-7
0.9	4.290e-9	7.510e-7	7.151e-7
1.0	6.849e-7	2.795e-7	2.141e-7

The rate of convergence is a critical metric for assessing the efficiency and effectiveness of numerical methods. Essentially, it quantifies how quickly a numerical solution approaches the exact solution as the number of iterations increases or as the discretization parameters are refined. The rate of convergence is calculated as follows:

$$\begin{aligned} ROC = \frac{\log {\dfrac{\Vert \varepsilon _1\Vert }{\Vert \varepsilon _2\Vert }}}{\log {\dfrac{M_1}{M_2}}}, \end{aligned}$$

where $\Vert \varepsilon _1\Vert ,\, \Vert \varepsilon _2\Vert$ are the maximum absolute error calculated at $M_1,\, M_2$, respectively.

**Table 7:** Rate of convergence of model (1) using DOMCM with $\nu =1$.

*M*	$\Vert \varepsilon _{\phi }\Vert$	$ROC_{\phi }$	$\Vert \varepsilon _{\chi }\Vert$	$ROC_{\chi }$	$\Vert \varepsilon _{\psi }\Vert$	$ROC_{\psi }$
9	4.384e-04	–	4.633e-04	–	2.528e-03	–
25	3.037e-05	2.61	4.073e-05	2.38	2.307e-04	2.34
30	8.596e-06	6.92	5.397e-06	11.09	2.219e-05	12.84
40	5.417e-07	9.61	3.012e-07	10.03	3.523e-06	6.40

Table 7 shows the maximum absolute error and the corresponding ROC for various values of *M*. On the other hand, the absolute error (**AE**) between the proposed method and Adams method is calculated and tabulated in Table 8. The absolute errors obtained by DOMCM are compared with those obtained by ANN-GA-SQPS^8^ where the results shows the accuracy of our technique.

**Fig. 2:**
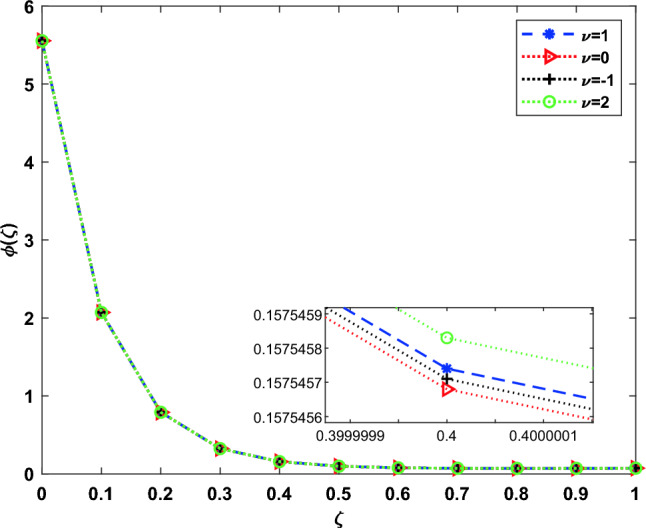
Comparison of the results of $\phi (\zeta )$ obtained by DOMCM at different values of $\nu$.

**Fig. 3:**
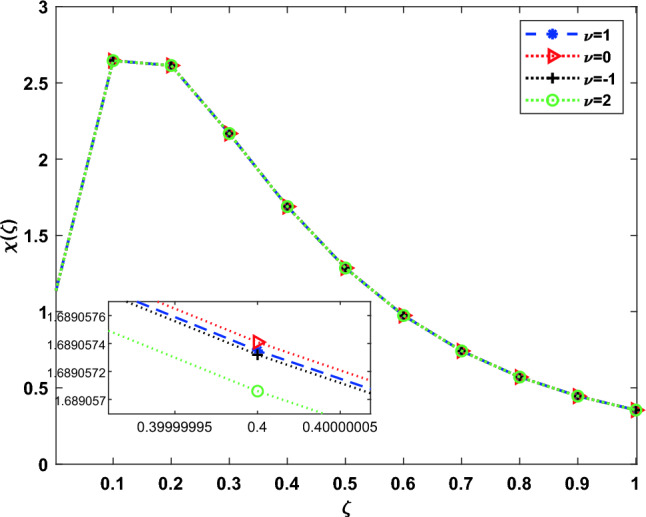
Comparison of the results of $\chi (\zeta )$ obtained by DOMCM at different values of $\nu$.

**Fig. 4:**
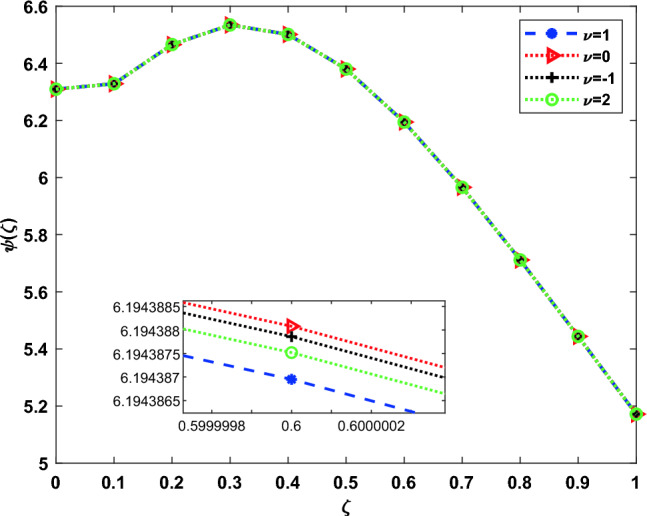
Comparison of the results of $\psi (\zeta )$ obtained by DOMCM at different values of $\nu$.

**Fig. 5:**
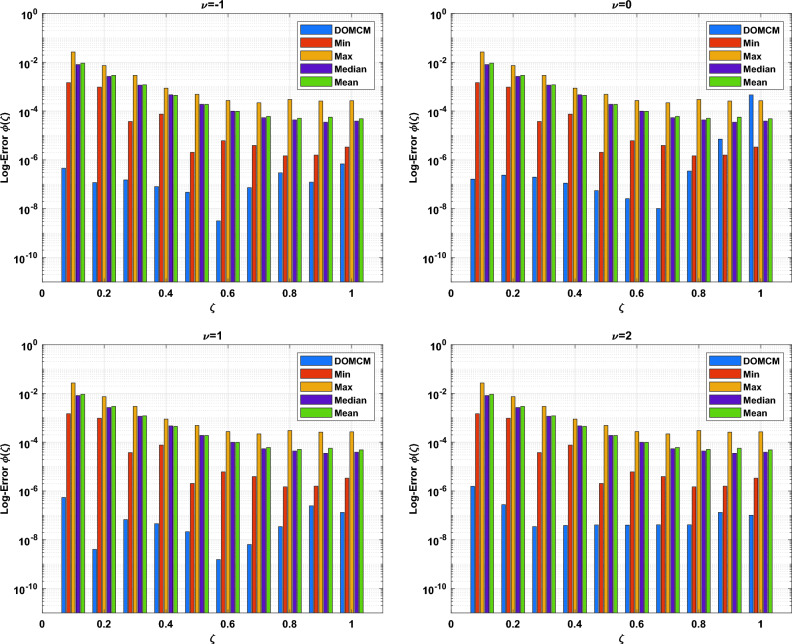
The comparison between AE of $\phi (\zeta )$ by DOMCM and Max., Min, Mean and Median of AE in^8^ for $\phi (\zeta )$.

**Fig. 6:**
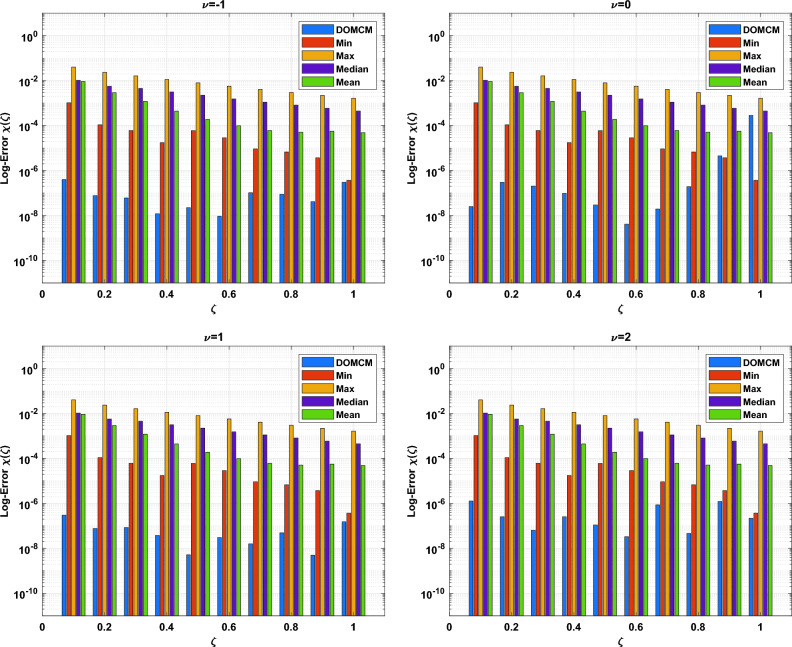
The comparison between AE of $\chi (\zeta )$ by DOMCM and Max., Min, Mean and Median of AE in^8^ for $\chi (\zeta )$.

**Fig. 7:**
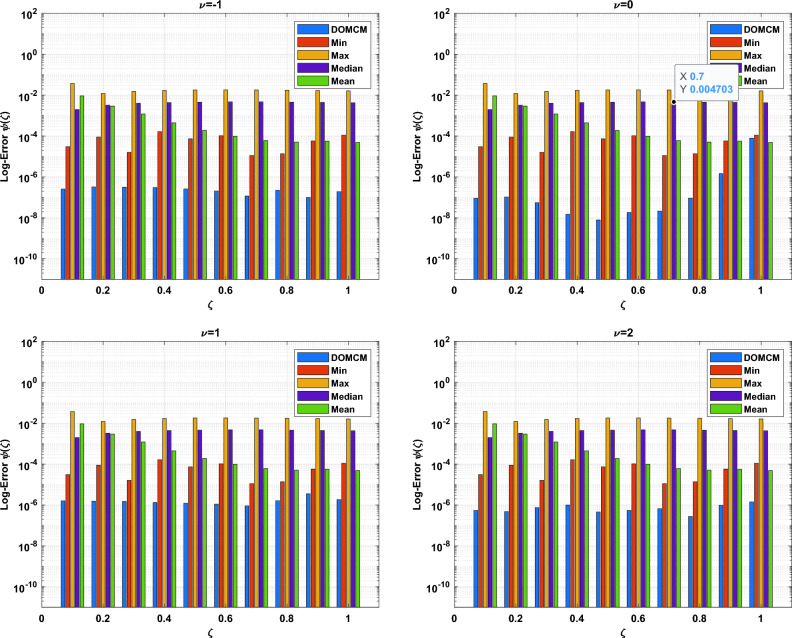
The comparison between AE of $\psi (\zeta )$ by DOMCM and Max., Min, Mean and Median of AE in^8^ for $\psi (\zeta )$.

The DOMCM absolute error is smaller then all the statistical values of the ANN-GA-SQPS algorithm (Mean, Median, Maximum and Minimum) as in Table 9 for the unknown function $\phi (\zeta )$, Table 10 for $\chi (\zeta )$ and Table 11 for $\psi (\zeta )$. To emphasize the accuracy of the proposed technique, the approximate solutions of the four cases of Dickson polynomials are plotted in Fig. 2 for $\chi (\zeta )$, Fig. 3 for $\phi (\zeta )$ and Fig. 4 for $\psi (\zeta )$. The figures show the high accuracy of the four cases included in Dickson collocation algorithm. The absolute errors obtained by the DOMCM are compared with the corresponding statistical values in^8^ in the form of bar charts for $\phi (\zeta )$ in Fig. 5, $\chi (\zeta )$ in Fig. 6 and $\psi (\zeta )$ in Fig. 7.

**Table 8:** The approximate solutions of the model (1-2) using Dickson collocation techniques for different values of $\nu$.

$\nu$	$\zeta$	$\phi (\zeta )$	AE[$\phi$]	$\chi (\zeta )$	AE[$\chi$]	$\psi (\zeta )$	AE[$\psi$]
$-1$	0.0	5.5555	3.722e-33	1.1111	9.441e-33	6.3096	2.729e-32
	0.1	2.07104896	4.583e-07	2.64591726	3.990e-07	6.32850630	2.582e-07
	0.2	0.79039206	1.184e-07	2.61443549	7.670e-08	6.46562824	3.245e-07
	0.3	0.32432968	1.520e-07	2.16821671	6.048e-08	6.53432802	3.134e-07
	0.4	0.15754571	8.038e-08	1.68905732	1.202e-08	6.50124205	3.007e-07
	0.5	0.09865198	4.692e-08	1.28614530	2.251e-08	6.38006772	2.605e-07
	0.6	0.07832328	3.184e-09	0.97467838	9.187e-09	6.19438786	2.044e-07
	0.7	0.07188731	7.318e-08	0.74212605	1.033e-07	5.96586950	1.168e-07
	0.8	0.07059141	2.971e-07	0.57104896	8.731e-08	5.71152520	2.249e-07
	0.9	0.07129632	1.232e-07	0.44591659	4.156e-08	5.44386839	1.005e-07
	1.0	0.07284520	6.843e-07	0.35449300	3.012e-07	5.17178119	1.901e-07
0	0.0	5.5555	0	1.1111	0	6.3096	0
	0.1	2.07104867	1.637e-07	2.64591769	2.526e-08	6.32850646	9.112e-08
	0.2	0.79039194	2.376e-07	2.61443572	2.996e-07	6.46562846	1.048e-07
	0.3	0.32432964	1.966e-07	2.16821685	2.060e-07	6.53432828	5.535e-08
	0.4	0.15754568	1.119e-07	1.68905741	9.708e-08	6.50124233	1.506e-08
	0.5	0.09865198	5.545e-08	1.28614535	2.957e-08	6.38006798	7.843e-09
	0.6	0.07832326	2.576e-08	0.97467839	4.156e-09	6.19438808	1.823e-08
	0.7	0.07188722	1.018e-08	0.74212614	1.940e-08	5.96586963	2.133e-08
	0.8	0.07059135	3.525e-07	0.57104924	1.935e-07	5.71152552	9.198e-08
	0.9	0.07130358	7.135e-06	0.44591202	4.525e-06	5.44386703	1.459e-06
	1.0	0.07330754	4.630e-04	0.35420979	2.835e-04	5.17170257	7.881e-05
1	0.0	5.5555	1.477e-32	1.1111	6.291e-33	6.3096	2.685e-31
	0.1	2.07104905	5.417e-07	2.64591736	3.012e-07	6.32850495	1.603e-06
	0.2	0.79039218	4.071e-09	2.61443549	7.643e-08	6.46562702	1.547e-06
	0.3	0.32432977	6.731e-08	2.16821673	8.429e-08	6.53432684	1.490e-06
	0.4	0.15754574	4.573e-08	1.68905735	3.774e-08	6.50124101	1.337e-06
	0.5	0.09865201	2.147e-08	1.28614532	5.174e-09	6.38006675	1.225e-06
	0.6	0.07832328	1.555e-09	0.97467836	3.042e-08	6.19438695	1.106e-06
	0.7	0.07188723	6.421e-09	0.74212614	1.598e-08	5.96586871	8.978e-07
	0.8	0.07059174	3.434e-08	0.57104900	4.894e-08	5.71152381	1.616e-06
	0.9	0.07129669	2.469e-07	0.44591655	4.969e-09	5.44386496	3.523e-06
	1.0	0.07284465	1.332e-07	0.35449315	1.534e-07	5.17177955	1.832e-06
2	0.0	5.5555	1.424e-31	1.1111	1.108e-30	6.3096	4.248e-31
	0.1	2.07105005	1.547e-06	2.64591637	1.290e-06	6.32850601	5.413e-07
	0.2	0.79039245	2.755e-07	2.61443516	2.543e-07	6.46562904	4.764e-07
	0.3	0.32432980	3.474e-08	2.16821658	6.402e-08	6.53432758	7.482e-07
	0.4	0.15754583	3.849e-08	1.68905706	2.558e-07	6.50124136	9.926e-07
	0.5	0.09865199	4.089e-08	1.28614521	1.097e-07	6.38006753	4.516e-07
	0.6	0.07832324	4.000e-08	0.97467836	3.341e-08	6.19438752	5.432e-07
	0.7	0.07188728	4.105e-08	0.74212529	8.645e-07	5.96586896	6.553e-07
	0.8	0.07059175	4.121e-08	0.57104910	4.627e-08	5.71152570	2.727e-07
	0.9	0.07129657	1.321e-07	0.44591776	1.219e-06	5.44386946	9.714e-07
	1.0	0.07284462	1.015e-07	0.35449352	2.181e-07	5.17177997	1.412e-06

**Table 9:** Comparison of the AE obtained by the Dickson collocation technique and ANN-GA-SQPS^8^ for $\phi (\zeta )$.

$\zeta$	AE-DOMCM				Min. AE	Max. AE	Med. AE	Mean AE
	$\nu =-1$	$\nu =0$	$\nu =1$	$\nu =2$				
0	3.722e-33	0	1.477e-32	1.424e-31	5.358e-08	2.993e-04	1.462e-06	1.128e-05
0.1	4.583e-07	1.637e-07	5.417e-07	1.547e-06	1.478e-03	2.679e-02	8.167e-03	9.244e-03
0.2	1.184e-07	2.376e-07	4.071e-09	2.755e-07	9.628e-04	7.407e-03	2.647e-03	2.925e-03
0.3	1.520e-07	1.966e-07	6.731e-08	3.474e-08	3.768e-05	2.931e-03	1.161e-03	1.203e-03
0.4	8.038e-08	1.119e-07	4.573e-08	3.849e-08	7.597e-05	8.823e-04	4.649e-04	4.439e-04
0.5	4.692e-08	5.545e-08	2.147e-08	4.089e-08	2.040e-06	4.877e-04	1.909e-04	1.895e-04
0.6	3.184e-09	2.576e-08	1.555e-09	4.000e-08	6.094e-06	2.733e-04	9.913e-05	9.877e-05
0.7	7.318e-08	1.018e-08	6.421e-09	4.105e-08	3.931e-06	2.199e-04	5.401e-05	6.067e-05
0.8	2.971e-07	3.525e-07	3.434e-08	4.121e-08	1.477e-06	3.030e-04	4.376e-05	5.080e-05
0.9	1.232e-07	7.135e-06	2.469e-07	1.321e-07	1.579e-06	2.602e-04	3.522e-05	5.631e-05
1.0	6.843e-07	4.630e-04	1.332e-07	1.015e-07	3.379e-06	2.669e-04	3.919e-05	4.864e-05

**Table 10:** Comparison of the AE obtained by the Dickson collocation technique and ANN-GA-SQPS^8^ for $\chi (\zeta )$.

$\zeta$	AE-DOMCM				Min. AE	Max. AE	Med. AE	Mean AE
	$\nu =-1$	$\nu =0$	$\nu =1$	$\nu =2$				
0.0	9.441e-33	0	6.291e-33	1.108e-30	1.736e-09	4.244e-04	2.329e-06	1.128e-05
0.1	3.990e-07	2.526e-08	3.012e-07	1.290e-06	1.048e-03	4.029e-02	1.042e-02	9.244e-03
0.2	7.670e-08	2.996e-07	7.643e-08	2.543e-07	1.088e-04	2.370e-02	5.677e-03	2.925e-03
0.3	6.048e-08	2.060e-07	8.429e-08	6.402e-08	6.056e-05	1.637e-02	4.509e-03	1.203e-03
0.4	1.202e-08	9.708e-08	3.774e-08	2.558e-07	1.753e-05	1.131e-02	3.163e-03	4.439e-04
0.5	2.251e-08	2.957e-08	5.174e-09	1.097e-07	6.013e-05	8.015e-03	2.243e-03	1.895e-04
0.6	9.187e-09	4.156e-09	3.042e-08	3.341e-08	2.874e-05	5.736e-03	1.533e-03	9.877e-05
0.7	1.033e-07	1.940e-08	1.598e-08	8.645e-07	9.208e-06	4.120e-03	1.108e-03	6.067e-05
0.8	8.731e-08	1.935e-07	4.894e-08	4.627e-08	6.709e-06	2.999e-03	8.142e-04	5.080e-05
0.9	4.156e-08	4.525e-06	4.969e-09	1.219e-06	3.716e-06	2.218e-03	5.919e-04	5.631e-05
1.0	3.012e-07	2.835e-04	1.534e-07	2.181e-07	3.688e-07	1.649e-03	4.483e-04	4.864e-05

**Table 11:** Comparison of the AE obtained by the Dickson collocation technique and ANN-GA-SQPS^8^ for $\psi (\zeta )$

$\zeta$	AE-DOMCM				Min. AE	Max. AE	Med. AE	Mean AE
	$\nu =-1$	$\nu =0$	$\nu =1$	$\nu =2$				
0.0	2.729e-32	0	2.685e-31	4.248e-31	1.188e-08	5.648e-05	1.904e-06	1.128e-05
0.1	2.582e-07	9.112e-08	1.603e-06	5.413e-07	3.035e-05	3.706e-02	1.976e-03	9.244e-03
0.2	3.245e-07	1.048e-07	1.547e-06	4.764e-07	8.904e-05	1.213e-02	3.256e-03	2.925e-03
0.3	3.134e-07	5.535e-08	1.490e-06	7.482e-07	1.602e-05	1.524e-02	4.006e-03	1.203e-03
0.4	3.007e-07	1.506e-08	1.337e-06	9.926e-07	1.639e-04	1.706e-02	4.357e-03	4.439e-04
0.5	2.605e-07	7.843e-09	1.225e-06	4.516e-07	7.330e-05	1.792e-02	4.544e-03	1.895e-04
0.6	2.044e-07	1.823e-08	1.106e-06	5.432e-07	1.032e-04	1.809e-02	4.708e-03	9.877e-05
0.7	1.168e-07	2.133e-08	8.978e-07	6.553e-07	1.111e-05	1.790e-02	4.703e-03	6.067e-05
0.8	2.249e-07	9.198e-08	1.616e-06	2.727e-07	1.368e-05	1.750e-02	4.524e-03	5.080e-05
0.9	1.005e-07	1.459e-06	3.523e-06	9.714e-07	5.761e-05	1.687e-02	4.434e-03	5.631e-05
1.0	1.901e-07	7.881e-05	1.832e-06	1.412e-06	1.102e-04	1.612e-02	4.260e-03	4.864e-05

## Conclusions

This work presents an in-depth study of the nonlinear hepatitis B viral model, utilizing the Dickson collocation method for numerical simulation. Our results indicate that the Dickson collocation method effectively encapsulates the intricate dynamics of HBV interactions, providing enhanced accuracy and efficiency compared to traditional numerical methods and artificial neural networks. We demonstrated the robustness of our method in many scenarios through comprehensive simulations, highlighting its suitability for intricate biological models. The results enhance our understanding of hepatitis B dynamics and underscore the Dickson collocation technique’s potential as a valuable tool in mathematical modeling. Future research may explore further applications of this method to diverse viral infections and dynamic systems, thereby advancing the discipline of mathematical biology and offering insights that could inform clinical strategies for viral disease treatment.

## Data Availability

All data generated or analysed during this study are included in this published article.
